# Primary resistance to alectinib in a patient with STRN‐ALK‐positive non‐small cell lung cancer: A case report

**DOI:** 10.1111/1759-7714.13983

**Published:** 2021-05-07

**Authors:** Kunyan Sun, Ligong Nie, Lin Nong, Yuan Cheng

**Affiliations:** ^1^ Department of Respiratory and Critical Care Medicine Peking University First Hospital Beijing China; ^2^ Department of Pathology Peking University First Hospital Beijing China

**Keywords:** alectinib, ALK rearrangement, crizotinib, non‐small cell lung cancer, STRN‐ALK

## Abstract

Anaplastic lymphoma kinase (ALK) rearrangements are drivers of a subset of non‐small cell lung cancer (NSCLC). The rapid progression of ALK inhibitors has significantly prolonged the progression‐free survival of patients with ALK gene‐sensitive mutations. However, the response of patients with rare ALK rearrangements to tyrosine kinase inhibitors remains unknown. Here, we report a rare case of striatin (STRN)‐ALK‐positive NSCLC showing primary resistance to first‐line therapy alectinib and limited clinical activity of crizotinib in the alectinib‐resistant setting.

## Graphical abstract

Here, we report a rare case of striatin (STRN)‐ALK‐positive NSCLC responding to crizotinib after the failure of alectinib. Our study highlights the heterogeneity in response of STRN‐ALK fusion to targeted drugs and provides clinical evidence of STRN‐ALK fusion responding to crizotinib.

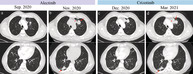

## INTRODUCTION

Anaplastic lymphoma kinase (ALK) rearrangements are reported in approximately 5% of patients with non‐small cell lung cancer (NSCLC).^1^ The striatin (STRN)‐ALK fusion is an extremely rare ALK rearrangement that was first described in 2013.^2^ In recent years, the development of ALK inhibitors has revolutionized the treatment of NSCLC and improved the overall and progression‐free survival (PFS) of ALK‐positive patients. However, the response to ALK inhibitors in patients harboring STRN‐ALK fusion remains unclear because of the paucity of cases. Here, we report a patient diagnosed with advanced lung adenocarcinoma with STRN‐ALK translocation who presented primary resistance to first‐line therapy alectinib.

## CASE REPORT

**FIGURE 1:**
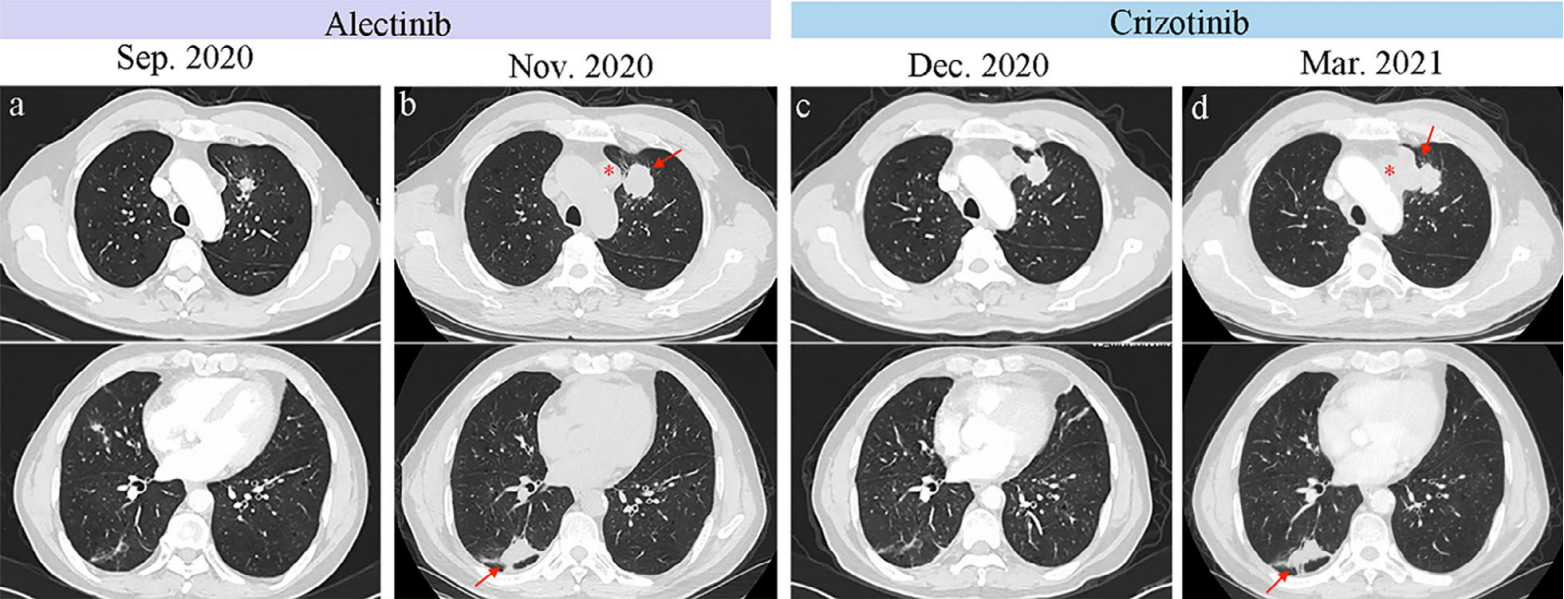
Images of chest computed tomography (CT) scans of the patient during the course of treatment. (a) Computed tomography scans during initial diagnosis. (b) One and a half months after initiation of alectinib. (c) One month after initiation of crizotinib. (d) Four months after crizotinib, the CT scan shows enlarged lymph nodes (asterisk) and an increase in size of the lung masses (arrow)

**FIGURE 2:**
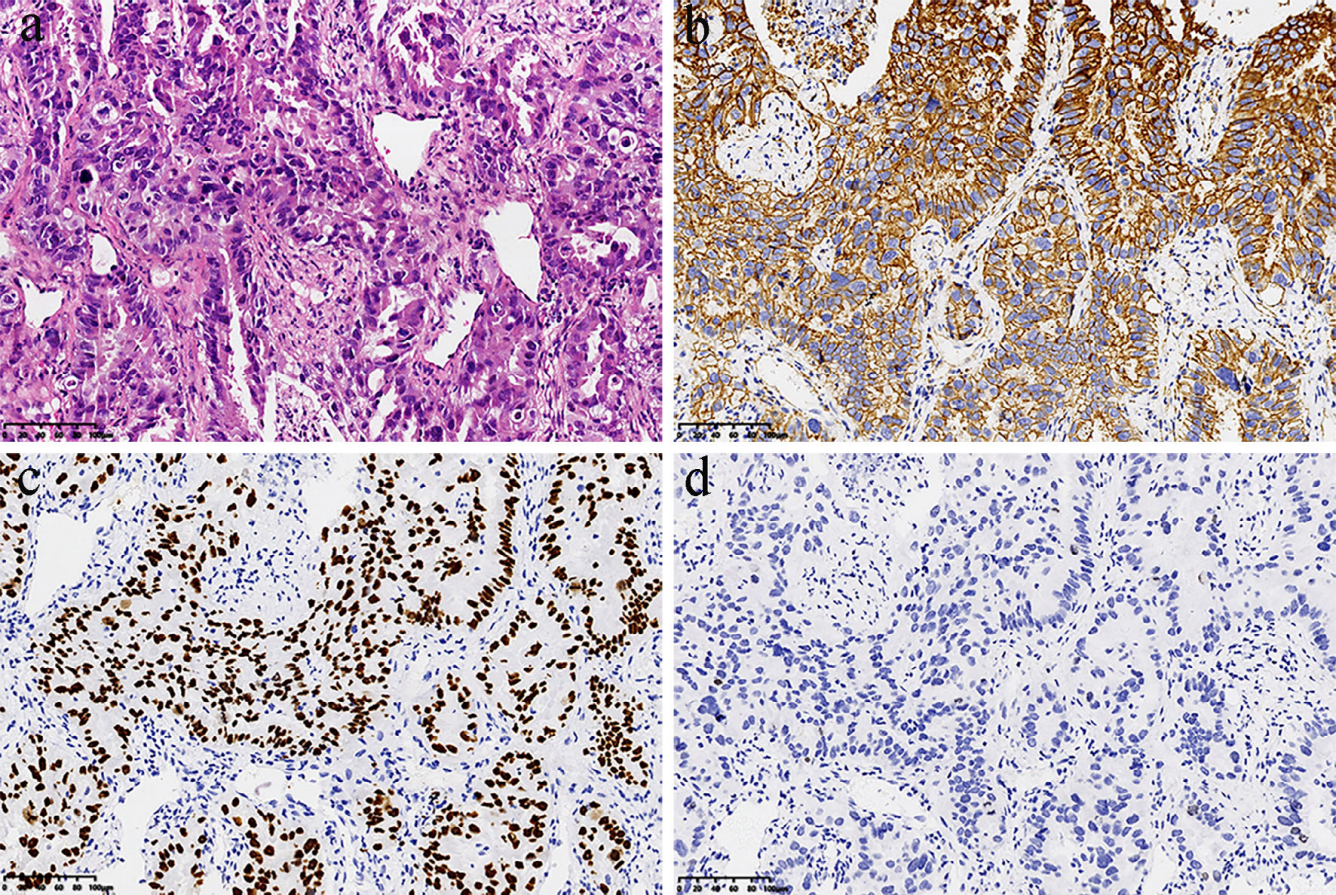
Histological findings. (a) Hematoxylin and eosin‐stained biopsy specimen (H&E, 200×). (b) Immunohistochemistry staining positive for napsin A (200×). (c) Immunohistochemistry staining positive for TTF‐1 (200×). (d) Immunohistochemistry staining negative for ALK (D5F3) (200×)

**FIGURE 3:**
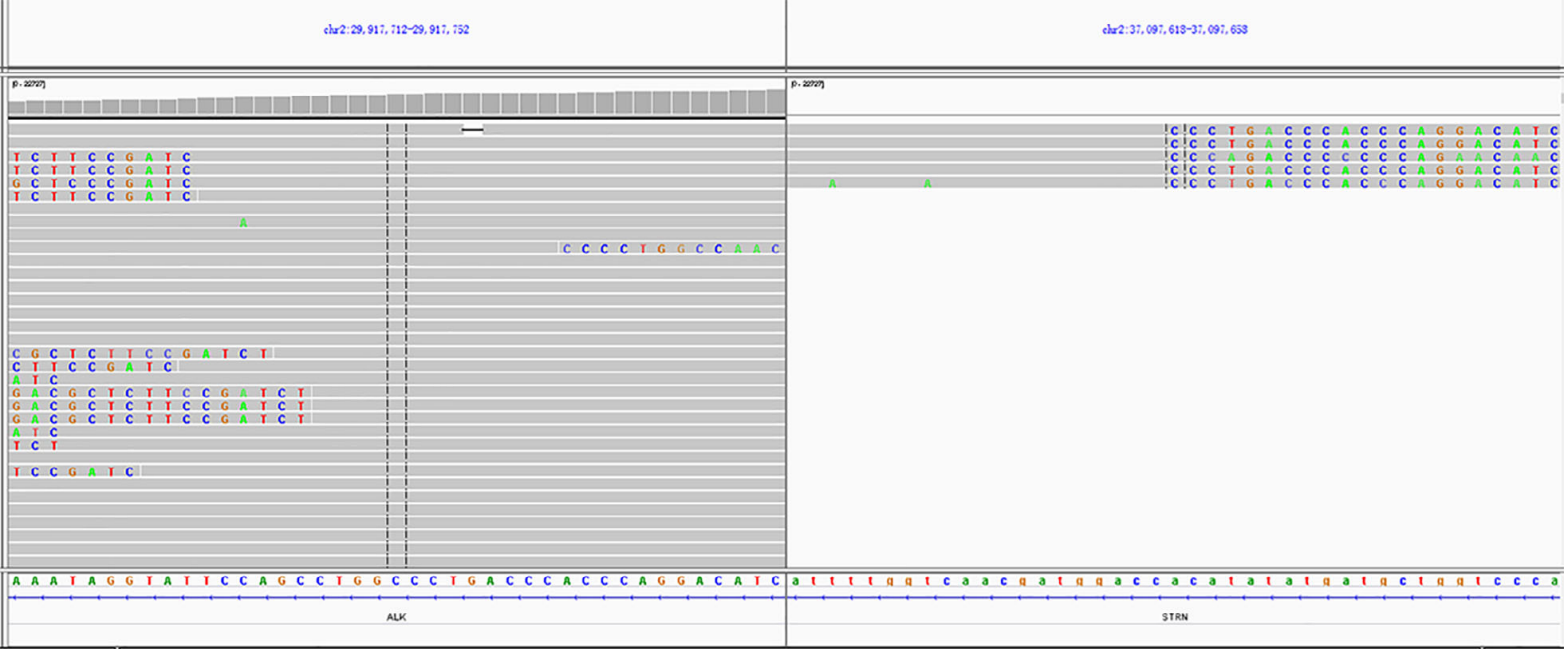
Next‐generation sequencing confirms STRN‐ALK fusion

A 65‐year‐old male with a 50‐year smoking history who presented with a chronic cough was admitted to our hospital in September 2020. Chest computed tomography (CT) demonstrated a mass in the upper lobe of the left lung, nodules in the lower lobe of the right lung, and bilateral hilar and mediastinal lymphadenopathy (Figure 1(a)). Positron emission tomography‐computed tomography revealed enlarged lymph nodes in the bilateral upper clavicle and bone metastasis at the L5 vertebrae. The patient then received a CT‐guided left lung biopsy, and pathological examination showed adenocarcinoma positive for Napsin A and transcription termination factor 1 (TTF‐1) (Figure 2(a)–(c)) (T2aN3M1b, stage IV A). Immunohistochemistry (IHC) staining for ALK (D5F3) was negative (Figure 2(d)). Next‐generation sequencing (NGS) of the biopsy specimen identified a rare STRN: exon3‐ALK: exon20 fusion (Figure 3) with a mutant allele frequency of 0.04%, and the specimen was positive for PIK3CA exon 2 mutation (Gly106Val, 53.63%), *KRAS* exon 2 mutation (Gly12Cys, 76.83%), and TP53 exon 8 mutation (Arg267His, 44.37%). No *EGFR* mutation or ROS‐1 rearrangement was detected by NGS. Alectinib was prescribed at a dose of 600 mg twice daily from September 2020. One and a half months later, the patient had disease progression with an increase in the size of the lung masses (Figure 1(b)). The treatment was switched to crizotinib (250 mg, twice a day) and a partial response was obtained after a one‐month follow‐up (Figure 1(c)). Our patient was in a stable condition on crizotinib treatment until March 2021 when the CT scan demonstrated significant disease progression with enlarged lymph nodes and increased size of the lung masses (Figure 1(d)). Upon progression on crizotinib, pemetrexed and carboplatin chemotherapy was started.

## DISCUSSION

STRN‐ALK fusion has been reported in multiple cancers including thyroid, colorectal, and renal cancer, and is related to aggressive features such as distant and lymph node metastasis.^3^, ^4^ The case we present here had the same breakpoint as described in previous studies.^5^, ^6^ The fusion represents an intrachromosomal translocation involving exons 1 to 3 of STRN and exons 20 to 29 of ALK within the short arm of chromosome 2, retaining the N‐terminal coiled‐coil domains of STRN and the intracellular region of ALK that contains the tyrosine kinase domain.^4^ Functionally, the coiled‐coil domains of STRN mediate constitutive activation of ALK kinase via ligand‐independent dimerization, which leads to the trigger of downstream signaling pathways and the formation of tumors.^3^

Alectinib has been previously reported to demonstrate significant prolongation in PFS in patients with untreated ALK‐positive NSCLC, and several crizotinib‐resistant ALK rearrangements could be inhibited by alectinib.^7^, ^8^ It is now the established first‐line treatment for advanced ALK‐positive NSCLC.^7^ In the literature review, only two cases choosing alectinib as the first‐line treatment of STRN‐ALK rearrangement have been reported. One of the patients responded to alectinib at first but progressed after three months .^5^ In contrast, the other case achieved an excellent response exceeding 19 months.^9^ Our study is the first report on primary resistance to alectinib in STRN‐ALK rearrangement. Mechanisms of alectinib resistance are mainly focused on acquired resistance which includes *ALK* mutations such as G1202R and I1171T/N/S, histological transformation, mesenchymal epithelial transition activation, HER2 gene amplification, and activation of bypass pathways.^10^, ^11^, ^12^, ^13^, ^14^, ^15^, ^16^ However, the mechanism of primary resistance to alectinib has been rarely reported. A preclinical study indicated ALK‐rearranged NSCLC cell lines concomitant with TP53 mutations were resistant to alectinib‐induced apoptosis.^17^ Gainor et al. analyzed repeat biopsies from post‐second‐generation ALK‐TKI treated patients, in whom TP53 mutations occurred most frequently.^10^ In the same study, one alectinib‐resistant specimen showed no *ALK* resistance mutations but a PIK3CA G106V mutation, which is considered as a gain‐of‐function mutation consecutively increasing AKT phosphorylation. These findings suggest that coexistence of TP53 and PIK3CA might result in intrinsic alectinib resistance in our patient.

The patient responded well to second‐line therapy crizotinib at first. One possible explanation is that alectinib demonstrates high selectivity and strong binding affinity to ALK, whereas crizotinib exhibits a relatively low affinity to ALK and targets multiple tyrosine kinases such as MET and ROS1.^18^ MET bypass signaling is associated with alectinib resistance in clinical settings but can be inhibited by crizotinib.^13^ The possibility that activation of bypass signals occurs in STRN‐ALK rearrangements cannot be eliminated. However, limited clinical activity of crizotinib with a PFS of four months was noted in our patient, whose PFS was lower than the other two cases demonstrating a favorable response to crizotinib, with a PFS of four years and over six months, respectively.^6^, ^19^ Our results suggest that patients with a lower abundance of mutations may benefit less regarding PFS. Additional studies are required to verify this hypothesis.

In conclusion, this is the first report of a NSCLC patient with STRN‐ALK rearrangement exhibiting primary resistance to alectinib; in addition, limited clinical activity of crizotinib in the alectinib‐resistant setting was observed. This highlights the heterogeneity of STRN‐ALK fusion in response to ALK inhibitors, and could be crucial to help guide therapeutic decisions to achieve maximum benefit in the era of personalized cancer therapy. Additionally, our study suggested that coexistence of TP53 and PIK3CA might be associated with intrinsic alectinib resistance. As the pathogenic role of STRN‐ALK rearrangement is not fully understood, further studies are warranted.

## CONFLICT OF INTEREST

The authors have no conflicts of interest to declare.
